# pH-Sensitive Fluorescent Marker Based on Rhodamine 6G Conjugate with Its FRET/PeT Pair in “Smart” Polymeric Micelles for Selective Imaging of Cancer Cells

**DOI:** 10.3390/pharmaceutics16081007

**Published:** 2024-07-30

**Authors:** Igor D. Zlotnikov, Alexander A. Ezhov, Elena V. Kudryashova

**Affiliations:** 1 Faculty of Chemistry, Lomonosov Moscow State University, Leninskie Gory, 1/3, 119991 Moscow, Russia; zlotnikovid@my.msu.ru; 2 Faculty of Physics, Lomonosov Moscow State University, Leninskie Gory, 1/2, 119991 Moscow, Russia; alexander-ezhov@yandex.ru

**Keywords:** pH marker, rhodamine 6G, tumor targeting, imaging, polymeric micelles

## Abstract

Cancer cells are known to create an acidic microenvironment (the Warburg effect). At the same time, fluorescent dyes can be sensitive to pH, showing a sharp increase or decrease in fluorescence depending on pH. However, modern applications, such as confocal laser scanning microscopy (CLSM), set additional requirements for such fluorescent markers to be of practical use, namely, high quantum yield, low bleaching, minimal quenching in the cell environment, and minimal overlap with auto-fluorophores. R6G could be the perfect match for these requirements, but its fluorescence is not pH-dependent. We have attempted to develop an R6G conjugate with its FRET or PeT pair that would grant it pH sensitivity in the desired range (5.5–7.5) and enable the selective targeting of tumor cells, thus improving CLSM imaging. Covalent conjugation of R6G with NBD using a spermidine (spd) linker produced a pH-sensitive FRET effect but within the pH range of 7.0–9.0. Shifting this effect to the target pH range of 5.5–7.5 appeared possible by incorporating the R6G-spd-NBD conjugate within a “smart” polymeric micelle based on chitosan grafted with lipoic acid. In our previous studies, one could conclude that the polycationic properties of chitosan could make this pH shift possible. As a result, the micellar form of the NBD-spd-R6G fluorophore demonstrates a sharp ignition of fluorescence by 40%per1 pH unit in the pH range from 7.5 to 5. Additionally, “smart” polymeric micelles based on chitosan allow the label to selectively target tumor cells. Due to the pH sensitivity of the fluorophore NBD-spd-R6G and the selective targeting of cancer cells, the efficient visualization of A875 and K562 cells was achieved. CLSM imaging showed that the dye actively penetrates cancer cells (A875 and K562), while minimal accumulation and low fluorophore emission are observed in normal cells (HEK293T). It is noteworthy that by using “smart” polymeric micelles based on polyelectrolytes of different charges and structures, we create the possibility of regulating the pH dependence of the fluorescence in the desired interval, which means that these “smart” polymeric micelles can be applied to the visualization of a variety of cell types, organelles, and other structures.

## 1. Introduction

Currently, fluorescent pH sensors are widely used for detecting and visualizing cellular structures or cell surface receptors in living cells [1,2,3,4]. However, modern applications, such as confocal laser scanning microscopy (CLSM), set additional requirements for fluorescent markers to be of practical use, namely, high quantum yield, low bleaching, minimal quenching in the cell environment, and minimal overlap with auto-fluorophores. There is an urgent need to develop methods for the selective visualization of tumor cells, macrophages (sometimes together with bacteria absorbed by macrophages), peroxisomes, and lysosomes characterized by their acidified environment.

In this study, we concentrate on the selective visualization of tumor cells. We focus on two key aspects of fluorescence-based selective imaging of cancer cells: (i) the development of a fluorescent marker that is selective for slightly acidic pH environments and (ii) the selective penetration of this marker into cancer cells.

There are pronounced morphological differences between cancerous and healthy cells, as well as their different microenvironments [5,6,7,8]. In the extracellular microenvironment of solid tumors, the pH is more acidic (approximately a pH of 6–6.5) (the Warburg effect) compared to the blood (approximately 7.4–7.5) [9]. In addition, the pH inside tumor cells is usually also significantly lower (~5.5–6.5) compared to that of normal cells [10]. Thus, it is necessary to obtain a fluorophore that shows drastic changes in fluorescence intensity in this pH range of 7.5–5.5.

R6G could be the perfect match for these requirements, but its fluorescence is not pH-dependent. We attempted to develop an R6G conjugate with its FRET or PeT pair that would grant it pH sensitivity in the desired range (5.5–7.5) and enable the selective targeting of tumor cells, thus improving CLSM imaging.

NBD (4-nitro-2,1,3-benzoxadiazole) derivatives [3,11,12,13] are widely used in biochemical research as a fluorescent lipid label for the visualization of membrane processes in living cells and model systems, for studying the transmembrane transfer of amines, for monitoring gene transfection, and as a fluorescent carbohydrate label for the visualization of cancer cells [13,14]. To obtain a fluorophore with improved solubility, NBD-Cl can be modified with amines [11,14]. Modified NBD or pyrene is mentioned in the literature as a pH-dependent fluorophore that demonstrates a fluorescence increase at physiological pH values [15,16]. However, pyrene-based fluorophores are not suitable for use in studying cells using optical microscopy techniques: the need to excite fluorescence in the ultraviolet (UV) region and the high autofluorescence of cells limit their application. Therefore, a pH sensor capable of excitation in the visible long-wavelength region is required. NBD-based fluorophores are characterized by relatively low molar extinction coefficients (approximately 12,000–31,000 M^−1^ × cm^−1^) and low fluorescence quantum yields (approximately 0.027 in water). We suggested that the combination of two fluorophores (covalent conjugate) could increase the quantum yield, solubility, pH sensitivity, and other properties of a pH-sensitive marker. Here, we could create a pH-sensitive fluorophore based on rhodamine 6G conjugated with NBD-spermidine for the selective imaging of cancer cells.

In fact, fluorescent molecules can be made pH-sensitive in several ways using different mechanisms [4]: photon-induced electron transfer (PeT) [17], Foerster resonance energy transfer (FRET) [1,18,19], self-aggregation [20], monomer–excimer transition (excited dimer, for example, for pyrene [21,22,23,24]), and structural changes in the fluorophore molecule [25].

The effect of PeT is characteristic of NBD modified by amine bases (morpholine, amines), changing the electron density at fluorophore in a pH-dependent way [2,3]. When the fluorophore is excited, the electron moves from the highest occupied molecular orbital (HOMO) to the lowest unoccupied molecular orbital (LUMO) with subsequent emission of a photon when the electron returns to the HOMO. In this work, the PeT mechanism is involved due to the modification of NBD by spermidine.

The second mechanism of pH sensitivity is realized due to the FRET phenomenon: in the case of NBD, the appropriate acceptor is rhodamine 6G (R6G). FRET occurs under the following conditions: (1) the overlap of the emission and excitation spectra of fluorescence of two fluorophore molecules and (2) a distance of 1–10 nm between the fluorophore molecules. A pH-labile linker (C=N, N=N, S–S) can be introduced into a bifunctional FRET fluorescent molecule—so that when the linker is cleaved, the FRET signal disappears. 

The third mechanism behind pH sensitivity is the structural changes in the fluorophore molecule when pH changes dye self-aggregation. Self-aggregation is characteristic of rhodamine 6G (R6G), FITC (fluorescein isothiocyanate), carbocyanine derivatives, and some other fluorophores [4]. A change in the acidity of the medium may cause structural changes in the fluorophore molecule, for example, with the opening of a five-membered cycle in modified-R6G [25], which would affect the fluorescence. 

When NBD is conjugated with rhodamine 6G via an oligoamine spacer (e.g., spermidine), all of these mechanisms could be engaged, resulting in a high degree of fluorescence sensitivity to pH. Here, we have synthesized a new conjugate NBD-spd-R6G based on R6G with its FRET or PeT pair NBD, linked by the oligoamine spermidine spacer (which acts as a spacer and also provides pH sensitivity due to its cationic properties) as a potential pH-sensitive fluorophore that responds to changes in the environment.

The important task in the targeted imaging of cancer cells is the specific delivery of a fluorescent marker to these cells. We have previously demonstrated that “smart” polymeric “Aikido-micelles” show high selectivity for tumor cells [24,26,27] due to their pH, thermal, and redox sensitivity while minimizing the accumulation of the fluorophore in healthy cells [10,28,29,30,31,32,33]. Additionally, fluorophore molecules accumulate most actively in the hydrophobic core of the micelles, presumably enhancing the dye aggregation, FRET [34,35,36], and PeT due to the convergence and concentration of fluorophores. Therefore, the R6G-spd-NBD pH sensor loaded into chitosan-based polymeric micelles, as we expect, could selectively penetrate into tumor cells, resulting in fluorescence activation in a slightly acidic environment to selectively image the cancer cells.

Here, we suggested a novel approach to selectively stain cancer cells compared to normal through two mechanisms: (i) a pH-sensitive probe in the range of 5.5–7.5 and (ii) polymeric “Aikido micelles” that selectively deliver a fluorescent pH marker (or drug) to the cancer cells. The combination of these two factors resulted in unprecedented sensitivity and specificity of the fluorescence probe. This approach has potential in vitro and in vivo studies to stain cancer cells and tissue and also in cancer diagnosis and treatment, making this research valuable for both fundamental and practical medical applications.

## 2. Methods

### 2.1. Reagents

NBD-Cl (4-chloro-7-nitro-1,2,3-benzaxadiazole) was purchased from Thermofisher Scientific (Carlsbad, CA, USA). Rhodamine 6G (R6G), spermidine (spd), 4-hydroxybenzaldehyde, chitosan oligosaccharide lactate 5 kDa (Chit5), and lipoic acid (LA) were obtained from Sigma-Aldrich (St. Louis, MO, USA). p-toluenesulfonic acid (TsOH), components of buffer solutions, NaOH, and HCl were produced by Reachim (Moscow, Russia). Buffer solutions: 0.02 M sodium borate buffer with pH of 10.5, 9.2, 8.3; 0.02 M sodium phosphate buffer with pH of 7.4, 6.0; 0.02 M sodium acetate buffer with pH of 5.0; 0.02 M sodium citrate buffer with pH of 3.5; and 0.01 M and 0.1 M HCl with pH of 2.0 and 1.0.

### 2.2. Synthesis of pH-Sensitive Fluorophore

The synthesis of the pH-sensitive fluorophore NBD-spd-R6G was carried out in 4 stages: (1) protection of amino groups of spermidine (spd), (2) nucleophilic substitution of chlorine in NBD for the open amino group of spermidine (spd), (3) removal of protection from amino groups, and (4) formation of an amide bond from the ester in R6G and the amino group of spermidine to obtain the final substance.

Stage 1. The samples of spd (40 mg, 0.24 mmol) and 4-hydroxybenzaldehyde (48 mg, 0.42 mmol) were mixed and dissolved in 5 mL of a mixture of sodium acetate buffer (0.02 M, pH = 5) and ethanol (80/20 *v*/*v*), followed by the addition of a catalytic amount of TsOH (5 mol%) and incubation with stirring for 2 h at 60 °C. Purification from unreacted impurities was carried out via dialysis against distilled water (1 h, cut-off weight of 500 Da). The product spd-N=CH-C_6_H_4_-OH (white powder, 50 mg, 0.206 mmol, yield of 75%) was isolated after freeze-drying at a temperature of −60 °C (Edwards 5, BOC Edwards, Burgess Hill, UK).

Stage 2. NBD-Cl was modified according to the methods described in [3,11,13]. spd-N=CH-C_6_H_4_-OH (42 mg, 0.173 mmol) was mixed with NBD-Cl (35 mg, 0.175 mmol), followed by dissolution in borate buffer solution (0.02M, pH 8.5) and incubation with stirring for 30 min in boiling water a water bath. NBD-spd-N=CH-C_6_H_4_-OH (yellow-orange flakes, 62 mg, 0.150 mmol, yield of 86%) was isolated similarly to above in stage 1.

Stage 3. NBD-spd-N=CH-C_6_H_4_-OH (55 mg, 0.133 mmol) was dissolved in 0.01 M HCl and incubated for 30 min at 50 °C, with subsequent chromatographic separation from the protective group. The NBD-spd product (39 mg, 0.127 mmol, 95% yield) was isolated after freeze-drying at a temperature of −60 °C.

Stage 4. NBD-spd (30 mg, 0.098 mmol) was mixed with R6G (48 mg, 0.10 mmol), followed by dissolution in ethanol and incubation for 24 h at 55 °C. Purification was carried out via dialysis against distilled water (12 h, cut-off weight of 500 Da). The product NBD-spd-R6G (55 mg, 0.074 mmol, yield of 76%, final yield in four stages of 46%) was isolated after freeze-drying at a temperature of −60 °C.

The degree of modification of spermidine amino groups was calculated in accordance with spectrophotometric titration (before and after modification) with 2,4,6-trinitrobenzenesulfonic acid (TNBS) in 0.02 M sodium borate buffer (pH 9.2). The number of primary titrated amino groups found by this method is as follows: (1) spd—2 per molecule; (2) spd-N=CH-C_6_H_4_-OH—1 per molecule; (3) 0 NH_2_– per NBD-spd-N=CH-C_6_H_4_-OH molecule; (4) NBD-spd—1 per molecule; (5) NBD-spd-R6G—1 per molecule.

### 2.3. Synthesis of Chit5-LA Amphiphilic Conjugate and Polymeric Micelles Loaded with Fluorophore Preparation

The synthesis of lipoic acid (LA)-modified chitosan (5 kDa, Chit5) was carried out as described by us earlier [37]. Chitosan LA-grafting degree was determined by spectrophotometric titration of amino groups using 2,4,6-trinitrobenzenesulfonic acid (by absorption at 420 nm). Chit5 was used as control sample.

Amphiphilic Chit5 polymer (1 mg/mL) was mixed with R6G, NBD-spd, or pH sensor R6G-spd-NBD (1 µM) in PBS (0.01 M, pH of 7.4), and the mixtures were then incubated at 37 °C for 1 h. Micelle samples were obtained via ultrasonic treatment of solutions (22 kHz) for 15 min with constant cooling in an ultrasonic device (Cole-Parmer Instrument, Vernon Hills, IL, USA). Micellar solutions were extruded (5-fold, 200 nm membrane, Avanti Polar Lipids). The free fluorophores were then separated by dialysis against PBS (with a cut-off mass of 6–8 kDa), and the degree of loading was then determined by absorbance.

### 2.4. Characterization of Synthesized Substances and Polymer Micelles with Loaded Fluorophores

#### 2.4.1. Fourier Infrared Spectroscopy

The FTIR spectra of the samples in suspension were recorded using a Bruker Tensor 27 spectrometer equipped with a liquid nitrogen-cooled MCT (cadmium–mercury telluride) detector. A total of 35 µL of samples was placed in a thermostatically controlled (22 °C) BioATR-II cell with a ZnSe element (Bruker, Bremen, Germany), and IR spectra in the range from 850 to 4000 cm^−1^ with a spectral resolution of 1 cm^−1^ were recorded. 70 scans were accumulated and averaged for each spectrum. Spectral data were processed using the Bruker Opus 8.2.28 program (Bruker, Bremen, Germany).

#### 2.4.2. NMR Spectroscopy

A total of 10–15 mg of the samples was dissolved in 0.7–1 mL of DMSO-d6. ^1^H and ^13^C NMR spectra of the solutions were recorded on a Bruker Avance DRX-500 spectrometer (Bruker, Bremerhaven, Germany) with an operating frequency of 500 MHz.

#### 2.4.3. Fluorescence Spectroscopy

Fluorescence emission and excitation were measured using a Varian Cary Eclipse fluorescence spectrophotometer (Agilent Technologies, Santa Clara, CA, USA) at temperatures of 22 and 37 °C. λ_excitation_ = 460 or 515 nm. λ_emission_ = 550 nm.

#### 2.4.4. Circular Dichroism Spectroscopy

Circular dichroism (CD) spectra were recorded on Jasco J-815 CD Spectrometer (JASCO, Tokyo, Japan) and were used to estimate the deacetylation degree in Chit5, which amounted to (95 ± 3%).

#### 2.4.5. Atomic Force Microscopy

Atomic force microscopy (AFM microscope NTEGRA II, NT-MDT Spectrum Instruments, Moscow, Russia) was used to visualize polymeric micelles based on grafted chitosan and compare them in terms of shape and size with non-modified chitosan.

#### 2.4.6. Dynamic Light Scattering

Dynamic Light Scattering (DLS) was used to measure the particle sizes and zeta potentials using a Zetasizer Nano S “Malvern” (Worcestershire, UK).

### 2.5. HEK293T, A875, K562, Macrophage Cells Cultivation

The embryonic kidney human epithelium HEK293T cell line, human melanoma A875 cell line, human immortalized myelogenous leukemia K562 cell line, and human macrophage cell line [38] were obtained from the Lomonosov Moscow State University Depository of Live Systems Collection (Moscow, Russia). Cells were grown in RPMI-1640 medium (Gibco, Thermo Fisher Scientific Inc., Waltham, MA, USA) supplemented with 5% fetal bovine serum (Capricorn Scientific, Ebsdorfergrund, Germany) and 1% Na-pyruvate (Paneco, Moscow, Russia) at 5% CO_2_/95% air in a humidified atmosphere at 37 °C.

### 2.6. Confocal Laser Scanning Microscopy (CLSM)

Cancer and normal cells (2 × 10^6^ cells/mL) were incubated with 1 µM of NBD-spd, R6G, or R6G-spd-NBD in a free or micellar (0.5 mg/mL Chit5-LA) form, followed by double washing and transfer to the wells of a 96-well plate. The cells were then imaged using a CLSM.

Fluorescence images were obtained using the confocal laser scanning microscope (CLSM) Olympus FluoView FV1000 equipped with both a spectral version scan unit with emission detectors and a transmitted light detector. CLSM is based on the motorized inverted microscope Olympus IX81. The excitation wavelength 488 nm (multiline Argon laser) and dry objective lens Olympus UPLSAPO 40X NA 0.90 were used for the measurements. Laser power, sampling speed, and averaging were the same for all image acquisitions. The scan area was 80 × 80 µm^2^. Fluorescence was collected using the emission windows set at 510–560 nm (green channel) and 560–800 nm (red channel). The signals were adjusted to the linear range of the detectors. Olympus FV10 ASW 1.7 software was used for acquisition of the images.

## 3. Results and Discussion

### 3.1. Synthesis and Characterization of the pH-Sensitive Fluorophore NBD-spd-R6G

#### 3.1.1. R6G-spd-NBD Synthesis and FTIR Spectroscopy Characterization

The modification of the NBD group by nucleophiles follows the S_N_Ar reaction mechanism. Therefore, NBD can be modified with amines or thiols. This work implements a variation of this reaction in a borate buffer, which is the most efficient method for execution and, more importantly, purification. Amine cross-linking and the nucleophilic addition of R6G’s NH_2_ group to the C=O of the ester group are key steps in this process.

**Figure 1:**
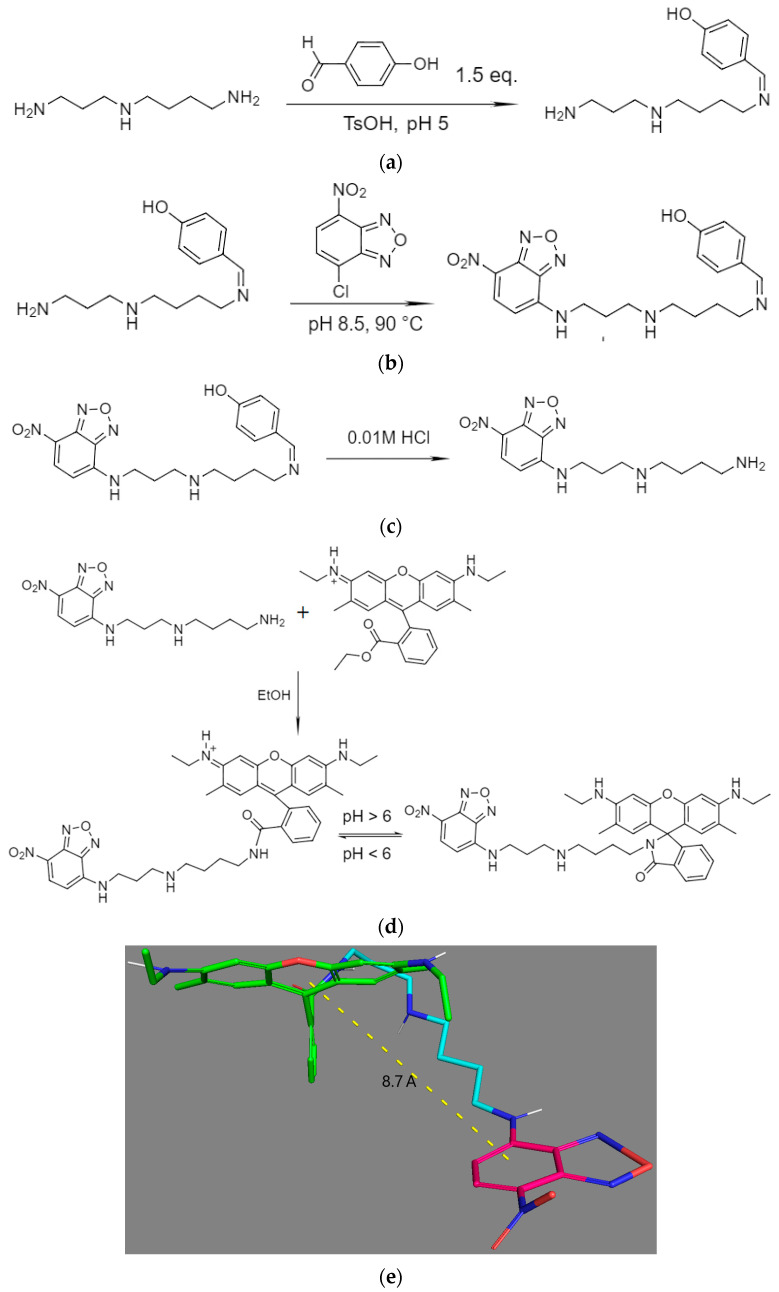
Synthesis scheme of the pH-sensitive fluorophore NBD-spd-R6G: (**a**) stage 1, (**b**) stage 2, (**c**) stage 3, and (**d**) stage 4. The products are a mixture of isomers (because spermidine is not symmetrical). (**e**) The 3D structure of the R6G-spd-NBD conjugates with the specified interplane distance. NBD—crimson, rhodamine—green, spacer spermidine—blue.

**Figure 2:**
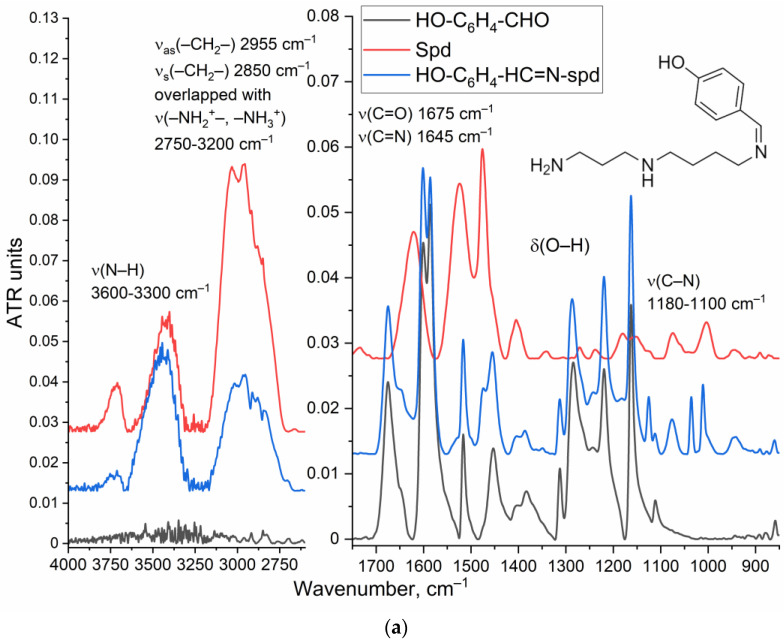

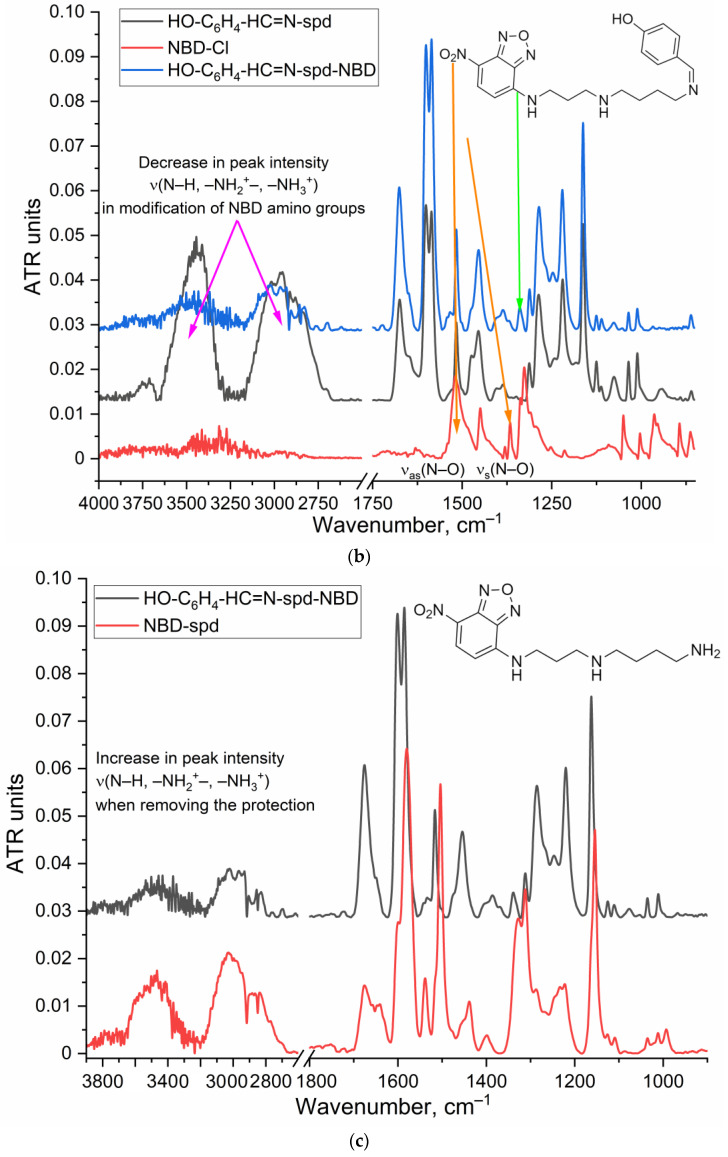

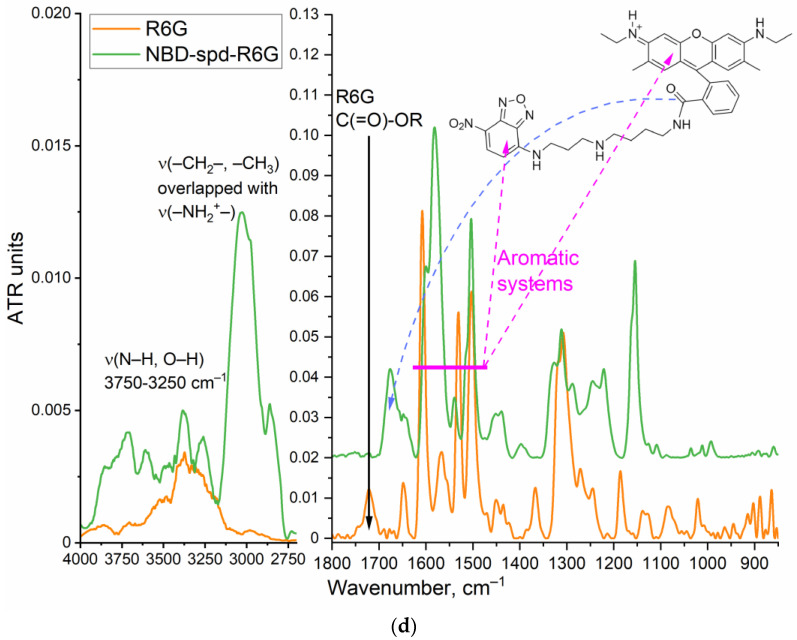
FTIR spectra of initial compounds, intermediate substances, and product of synthesis of pH-sensitive fluorophore R6G-spd-NBD: (**a**) stage 1, (**b**) stage 2, (**c**) stage 3, (**d**) stage 4. Aqueous solutions. T = 22 °C.

The synthesis of NBD-spd-R6G was carried out in four stages (Figure 1). In the first stage, the primary NH_2_ group (1 equivalent based on the initial quantities taken) of spd is protected with 4-hydroxybenzaldehyde HO-C_6_H_4_-CHO by the formation of a Schiff base HO-C_6_H_4_-HC=N-spd. In the FTIR spectrum (Figure 2a), the intensity of the band at 3200–2750 cm^−1^ decreases, corresponding to oscillations of N–H protonated amino groups, which indicates the modification of amino groups. The formation of the Schiff base is confirmed by the appearance of a peak at 1645 cm^−1^ (C=N). According to the data of spectrophotometric titration (Section 2), only one amino group of spermidine was modified in the first stage.

In the second stage, protected spermidine HO-C_6_H_4_-HC=N-spd reacted with NBD-Cl via the S_N_Ar reaction through the formation of the Meisenheimer complex [13]. In the FTIR spectra (Figure 2b), a decrease in the peaks of amino group fluctuations is observed due to modification by NBD. Bands corresponding to the fluctuations of the N–O nitro group (1365, 1520 cm^−1^), as well as to the aromatic system of NBD (δ(Carom–H) 1120–860 cm^−1^, ν(C=C) 1450 cm^−1^ and an overlapping peak of 1520 cm^−1^), are also observed.

In the third stage, the protection of spermidine amino groups was removed in an acidic environment with the formation NBD-spd, which is reflected in an increase in the intensity of N–H peaks (3600–330 cm^−1^, 3100–2750 cm^−1^) in the FTIR spectra (Figure 2c). As a result, the peaks of the aromatic aldehyde system (1570–1610 cm^−1^) disappeared.

The fourth stage—the crosslinking of NBD-spd with rhodamine 6G (R6G) occurring via the formation of an amide bond from an ester and an amine—is confirmed by the disappearance of the peak of the C=O ester group in R6G (1720 cm^−1^), the appearance of the peaks of amide bond (ν(C=O) 1680–1640 cm^−1^, δ(N–H) 1580 cm^−1^), and an increase in peak intensities ν(C–H) 3000–2800 cm^−1^, ν(C–N) 1155 cm^−1^ (Figure 2d). Thus, a compound containing two fluorophores (NBD and R6G) on a spd-spacer was obtained.

#### 3.1.2. NMR Spectroscopy for R6G-spd-NBD Characterization

**Figure 3:**
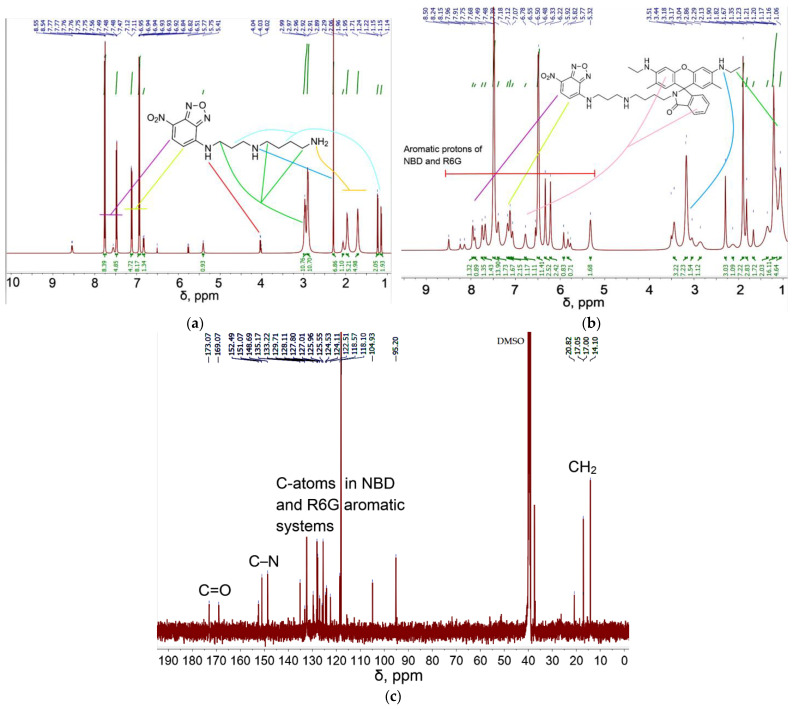
NMR spectra of (**a**) NBD-spd ^1^H, (**b**) NBD-spd-R6G ^1^H, (**c**) NBD-spd-R6G ^13^C. T = 25 °C. DMSO-d_6_, 500 MHz.

FTIR spectroscopy provides valuable information about functional groups, and complementary data of more detailed resolution can be obtained using NMR spectroscopy. Figure 3 shows the ^1^H and ^13^C NMR spectra of the products of the third and fourth stages of synthesis. For NBD-spd (Figure 3a), the most characteristic proton peaks of the aromatic system are 7.7–7.5 ppm (–CH–C(NO_2_)–) and 7.2–6.8 ppm (–CH–CH–C(NR)–) and peaks in the spacer spermidine of 1.1–3.0 ppm, which is consistent with the literature data for the initial components and similar compounds [14,39,40,41].

In the ^1^H NMR spectrum of NBD-spd-R6G, peaks appear in the range of 6–8 ppm, corresponding to protons of the aromatic R6G system. In the ^13^C NMR spectrum of NBD-spd-R6G, peaks in the range of 118–153 ppm corresponding to carbon atoms in the aromatic systems R6G and NBD, 14–21 ppm (CH_3_, CH_2_ groups), as well as peaks 169 and 173 ppm corresponding to the amide C atom for two equilibrium structures are observed (Figure 1). Thus, NMR spectroscopy confirms the formation of target products, but IR spectroscopy turned out to be the most informative.

#### 3.1.3. “Aikido Micelles” with pH Sensor: Synthesis, Properties, and Characterization

**Figure 4:**
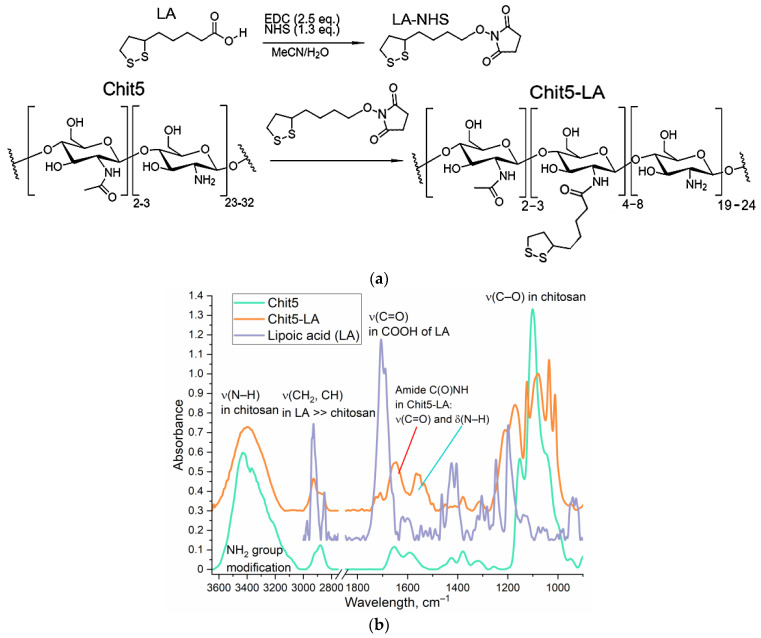

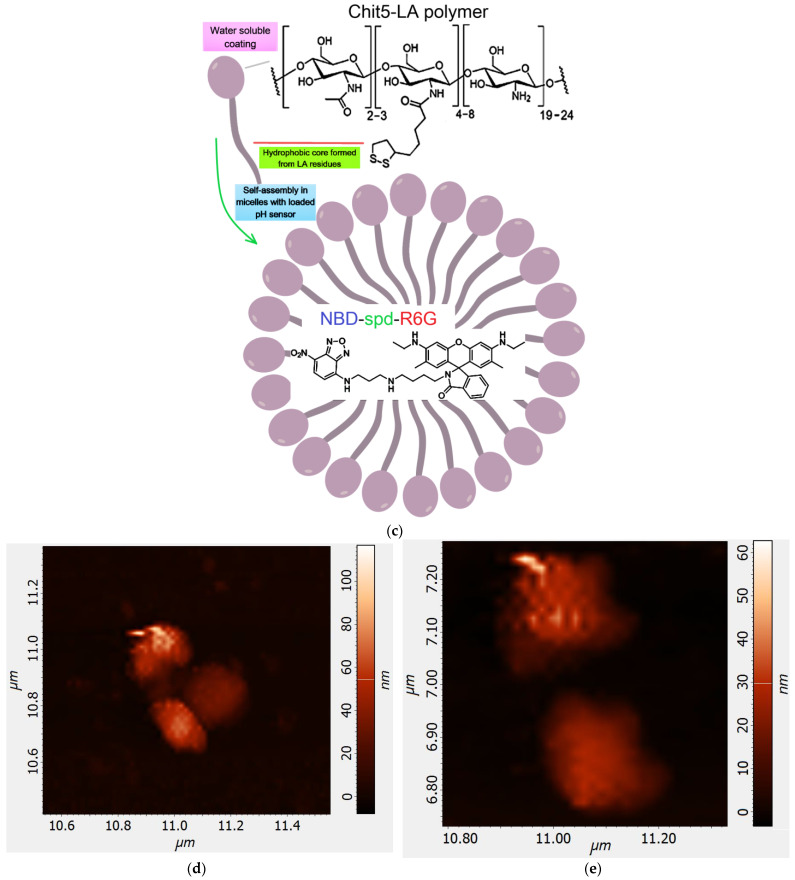

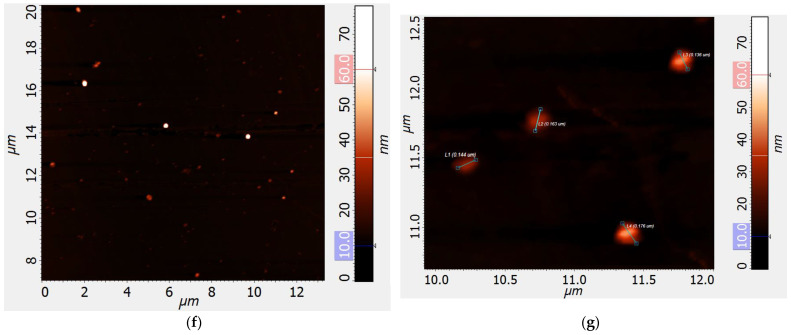
(**a**) Synthesis scheme of the amphiphilic micelle-forming conjugate Chit5-LA. (**b**) FTIR spectra Chit5, Chit5-LA, and lipoic acid (LA) in solid phase. T = 22 °C. (**c**) A schematic representation of the micelles formed by Chit5-LA with a loaded pH sensor R6G-spd-NBD. (**d**,**e**) Atomic force microscopy images of empty Chit5-LA. (**f**,**g**) Atomic force microscopy images of Chit5-LA micelles loaded with pH sensor R6G-spd-NBD.

The synthesis of chitosan 5 kDa (Chit5, deacetylation degree 95 ± 3%), grafted with lipoic acid residues, was carried out using the carbodiimide technique (Figure 4a). Conjugate Chit5-LA forms polymeric micelles with an average size of 170–250 nm. The characterization of modified chitosan with lipoic acid residues (Chit5-LA) was carried out by us earlier; the NMR spectra are presented in the Figure S1 [26]. Figure 4b shows FTIR spectra of chitosan 5 kDa (Chit5), lipoic acid (OA), and its conjugate of Chit5-LA. The spectra of LA and the conjugates contain characteristic bands corresponding to valence oscillations of CH_2_ groups in acid residues (2980–2850 cm^−1^). When Chit5 is modified with LA residues, a C(=O)NH amide group is formed from the COOH group, resulting in a decrease in the intensity of a peak at 1710 cm^−1^ and the appearance of two peaks at 1660 and 1560 cm^−^¹ (Figure 4b). Some of the amino groups of chitosan have been converted to amide crosslinks, leading to a decrease in the intensity of NH oscillations at 3600–3200 cm^−1^. Chitosan grafting with fatty acids causes a change in the shape and structure of oscillations in the C–O–C bonds (1200–1000 cm^−1^) in the polymer chain of chitosan, with the two-peak spectrum becoming multi-peaked due to differences in the hydrophobicity of microenvironments of glucosamine residues. Thus, the structure of polymer chitosan micelles was confirmed using FTIR spectroscopy.

A schematic representation of the micelles formed by Chit5-LA with a loaded pH sensor is shown in Figure 4c. AFM images of empty Chit5-LA micelles (Figure 4d,e) are shown in comparison with AFM images of Chit5-LA micelles loaded with the pH sensor R6G-spd-NBD (Figure 4f,g). Empty micelles are nanoparticles with an average diameter of approximately 150–250 nm, while the shape of these particles is loose and not completely spherical. Loading aromatic R6G-spd-NBD molecules into the hydrophobic core of the micelles leads to a compactization of the micelles, resulting in a decrease in the size of the nanoparticles to approximately 130–180 nm. Additionally, the particles are more dense and spherical.

The physico-chemical properties of the amphiphilic conjugate Chit5-LA and the micelles formed by it are presented in Table 1. The mass fraction of loaded fluorophores R6G, NBD-spd, and R6G-spd-NBD in micelles was 12, 15, and 10%, respectively (Table 1).

### 3.2. pH and Thermal Dependence of NBD-spd-R6G Fluorescence in Comparison with Free NBD and R6G in Aqueous Solution and in Micellar Systems

The pH sensitivity of the fluorophore, as discussed above, can be realized by a number of mechanisms: photon-induced electron transfer (PeT), the effect of resonant fluorescence energy transfer (FRET), the effect of energy transfer associated with self-aggregation, and structural changes in the fluorophore molecule when the pH changes. For the NBD-spd-R6G conjugate, FRET and cycle opening in R6G (Figure 1d) are realized with a decrease in pH in the microenvironment of the label due to protonation of the amino group of both spd and R6G with the opening of the ester lactone. 

**Figure 5:**
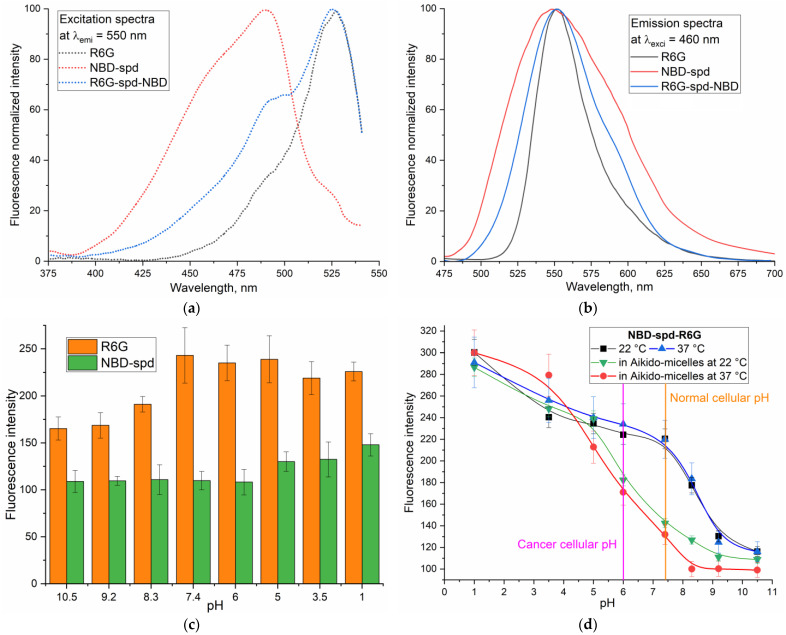
(**a**) Fluorescence excitation spectra of Rhodamine 6G (R6G), NBD, and R6G-spd-NBD at a fixed emission wavelength of 550 nm are shown. (**b**) Fluorescence emission spectra of R6G, NBD, and R6G-spd-NBD are shown at a fixed excitation wavelength of 460 nm. PBS (0.01 M, pH 7.4). T = 37 °C. (**c**) The fluorescence-emission intensity of R6G and NBD-spd is plotted against pH. NBD-Cl does not fluoresce. (**d**) The intensity of NBD-spd-R6G fluorescence emission is plotted against pH, temperature, and shape (simple or poly-dimensional Aikido micelles based on Chit5-LA). The dotted line highlights the range of the sharp pH transition of the sensors.

Figure 5a,b show the excitation and emission fluorescence spectra of NBD-spd-R6G conjugate (pH sensor) in comparison with NBD-spd and R6G. R6G is characterized by a maximum wavelength of excitation of 525 nm, a maximum wavelength of emission of 550 nm, an extinction coefficient of 116,000 M^−1^ × cm^−1^, and a quantum yield of 0.95. NBD-spd is characterized by the following parameters: a maximum wavelength of excitation of 488 nm, a maximum wavelength of emission of 545 nm, and an extinction coefficient of 35,000 M^−1^ × cm^−1^ (and 22,000 M^−1^ × cm^−1^ for NBD). For the conjugate R6G-spd-NBD, the fluorescence excitation region shifted toward short wavelengths in comparison with free R6G, while the peak of fluorescence emission widens, as a result of the FRET effect NBD→R6G, is realized with an efficiency of about 38% (since the fixed interplane distance R6G–NBD is 8.7 A—Figure 1e). NBD-spd is an excellent fluorophore donor for R6G. On the other hand, lasers with wavelengths of 488 and 515 nm are usually available in confocal microscopy in regions of 400–530 nm. The 488 nm laser excites NBD-spd and the pH sensor NBD-spd-R6G. At the same time, the 515 nm laser excites R6G and the pH sensor without the involvement of the FRET process. Therefore, we can monitor both the FRET phenomenon, which occurs between NBD and R6G in the NBD-spd-R6G pH sensor, and the fluorescence of R6G, with a minimal contribution from FRET, as a control for the pH-dependence of fluorescence.

Figure 5c illustrates the dependency of fluorescence intensity from pH for NBD and R6G. Since NBD-Cl exhibits weak fluorescence, a modified form, NBD-spd, was used to control the effects of the conjugation of NBD and R6G on pH sensitivity. NBD-spd shows a slight increase in fluorescence intensity of approximately 20% when the pH decreases from 6 to 2. In other words, no significant pH-dependent pattern of fluorescence is observed. It has been reported in the literature that for both NBD and the modified NBD analog, a pH-dependent fluorescent pattern is not typically observed [2]. On the other hand, R6G shows pH-dependent fluorescence in the pH 7–9 region, which is probably due to the protonation of the amino group R6G with the opening of the ester lactone (Figure 1d): the fluorescence of R6G increases significantly due to the opening of its five-membered ring, which enhances fluorescence intensity (Figure 5c). However, fluorescence ignition occurs up to 50% in a wide range of changes in the acidity of the medium, which limits the applicability and does not fit the pH sensor criterion [2]. In the case of individual fluorophores, there is a minimal change in fluorescence when the pH shifts from 7.4 to 6.0. Conversely, for the NBD-spd-R6G conjugate, which will be discussed later, this effect is more pronounced.

The main objective of this work is to develop a pH sensor that exhibits a significant change in fluorescence when the environment transitions from neutral to slightly acidic. In particular, to visualize cancer cells, the target pH sensor must have high sensitivity in the pH range of 5.5–7.5, which is achieved for the R6G-spd-NBD label, where the intensity of rhodamine fluorescence in a slightly acidic medium is increased relatively weak for the non-micellar form and strong for the micellar form (Figure 5d, Table 2).

Apparently, for the R6G-spd-NBD label, fluorescence strongly depends on pH in the pH range of 7.5–9.5 with a coefficient of ~50% fluorescence change/unit pH (Table 2). At the same time, in the target pH range of 7.5–6, the signal increases up to 6%—this is not enough to selectively visualize cancer cells. There is also no significant effect within a given pH range as the temperature varies: with an increase in temperature from 22 to 37 °C, the pH sensitivity for the R6G-spd-NBD label increases only slightly. So, the pH sensitivity effect is achieved in more alkaline conditions (Figure 5d—blue and black curves).

To achieve pH-dependent fluorescence in the target pH range of 7.5–6, it is necessary to apply the special approach. For example, we have recently shown the pH and thermosensitive behavior of polymer micelles, which may play in favor of shifting the pH of the transition toward values of 6–7.5. Thus, we propose an original solution—to use micellar formulations of the R6G-spd-NBD label. Recently, we have developed “Aikido micelles” (chitosan–lipoic acid) that selectively deliver drugs to cancer cells but almost do not interact with the normal ones [26,37]. Polymeric micelles based on chitosan and lipoic acid (Chit5-LA) exhibit selective targeting toward tumor cells due to their sensitivity to pH, temperature, and redox conditions while minimizing the accumulation of the drug in healthy cells. Inspired by our findings, we realized that the use of polymer micelles would increase the effectiveness of the pH sensor studied here in terms of the selective imaging of tumor cells.

Apparently, in micellar systems, the excitation of R6G shifts to longer wavelengths, resulting in overlap with the emission spectrum of NBD (Figure 5a,b), enhancing FRET and total emission by 20–30% in the conjugate. For the micellar formulation of the pH sensor R6G-spd-NBD, a sharp sensitivity to a slightly acidic environment is achieved (Figure 5d, Table 2): ~40% fluorescence change/unit pH. At the same time, in comparison with free R6G-spd-NBD, the pH sensitivity for the micellar formulation is achieved in the desired pH range of 6–7.5—the fluorescence intensity increases up to 30%. An increase in temperature from 22 to 37 °C in the case of the micellar form of R6G-spd-NBD (compared with a simple one) expands the pH range in which a sharp change in fluorescence is observed with increased sensitivity (Figure 5d).

In comparison to the literature, pH sensors discussed above (pyrene- or NBD-based) R6G-spd-NBD have a comparable level of sensitivity and magnitude of fluorescence change: [3] presents a pH sensor based on NBD (NBD-diamine-morpholine) with a ~52% fluorescence change/unit pH in the pH range of 3.4–4.8. The sharpness of the transition for the literary sample is comparable to our pH sensor, but it was not achieved in the target pH range for tumor labeling. The proposed micellar formulation has the advantage of the selectivity to changes in fluorescence under slightly acidic conditions and at physiological temperatures (Table 2). Therefore, we expect that due to the use of “Aikido micelles”, the R6G-spd-NBD pH sensor will demonstrate high selectivity toward tumor cells (in comparison to normal cells) and therefore may be utilized for their visualization. This is a novel approach that has not been previously described.

Interestingly, control “poor-conjugate” NBD-R6G that was synthesized without the use of protective groups (stages 1–3) demonstrates a non-bright pH-dependent fluorescence (Figure S2). Thus, the use of protective groups for the linker makes it possible to obtain a stoichiometric conjugate and achieve good pH sensitivity for R6G-spd-NBD. 

### 3.3. Confocal Laser Scanning Microscopy Imaging of the Effect of pH Labeling on Cancerous And Normal Cells

pH values are the ones of key differences between cancerous and healthy cells: in the extracellular environment of solid tumors, the pH = 6–6.5 is more acidic than in blood (pH = 7.4) [9]. The pH inside tumor cells (~5.5–6.5) is also lower than in normal cells. To perceive these differences and realize the possibility of more selectively staining cancer cells, it is necessary to use a fluorophore that changes its fluorescence intensity when the pH changes between 7.4 and 5.5. Therefore, we test our hypothesis that the pH sensor R6G-spd-NBD in “Aikido micelles” is sensitive to the cell type: normal/cancerous.

**Figure 6:**
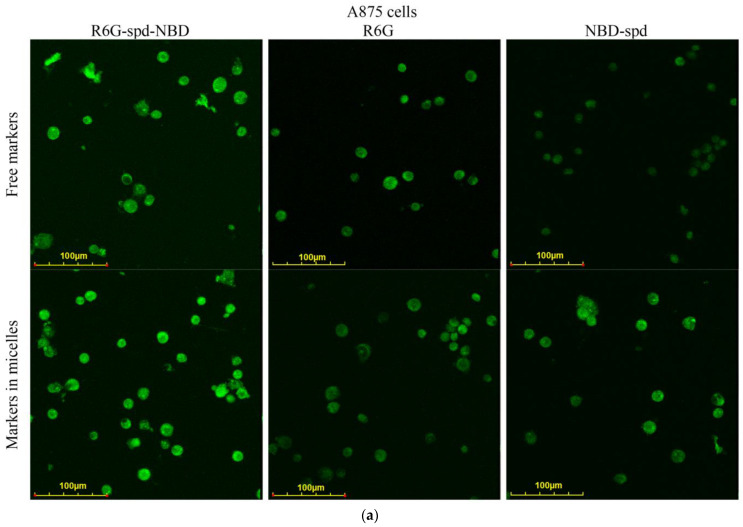

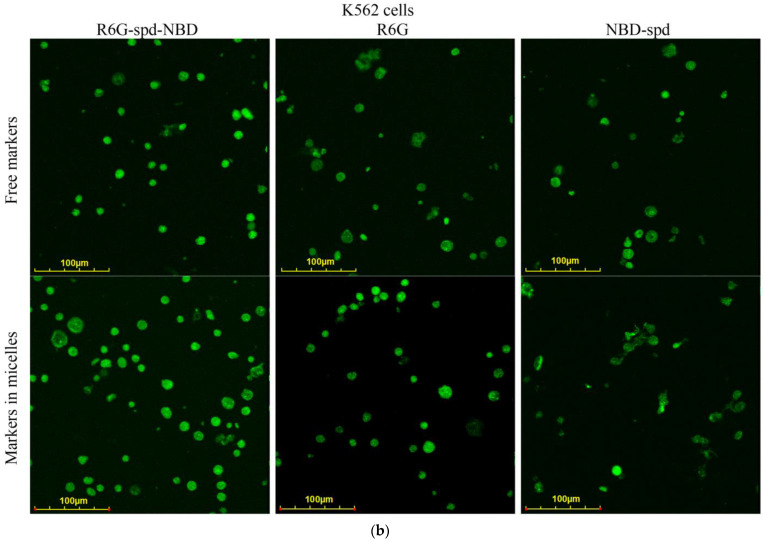

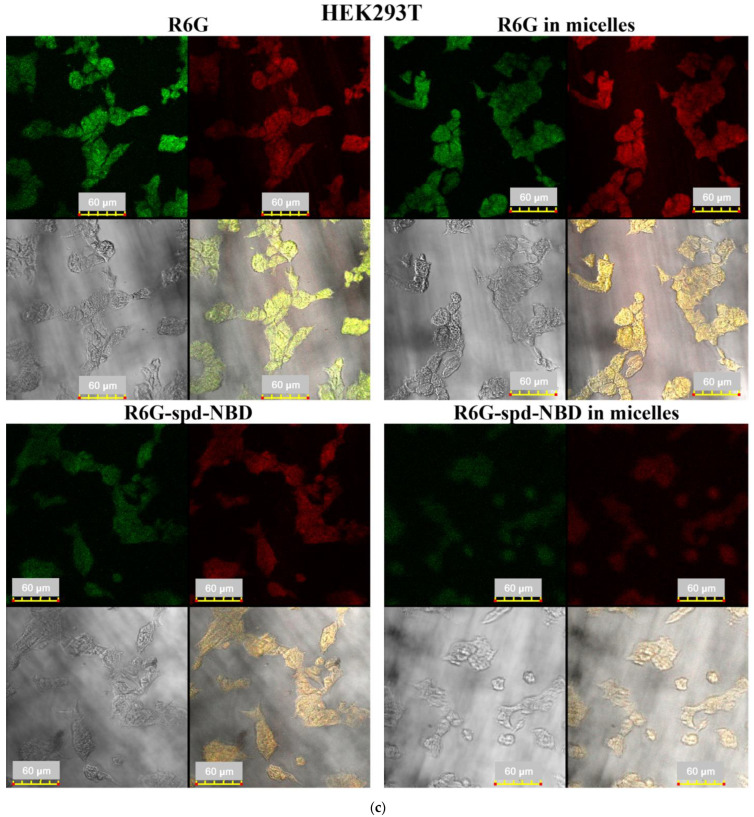
Confocal laser scanning microscopy images of (**a**) A875 cancer cells and (**b**) K562 cancer cells, labeled with NBD-spd, R6G, or R6G-spd-NBD (1 µg/mL for all markers). λ_exci_ = 488 nm, λ_emi_ = 510–560 nm (green). The scale segment is 100 µm. (**c**) Confocal laser scanning microscopy images of HEK293T normal cells labeled with R6G or R6G-spd-NBD (1 µg/mL for all markers). λ_exci_ = 488 nm, λ_emi_ = 510–560 nm (green), λ_emi_ = 560–800 nm (red). The green and red channels are shown, as well as a brightfield and merge. The scale segment is 60 µm. “Micelles” means Chit5-LA polymeric micelles. C_mic_ = 0.1 mg/mL.

In this work, we found that Aikido micelles greatly enhance the penetration of the dye into tumor cells (Figure 6); at the same time, micelles also reduce fluorophore penetration into healthy cells. To specify the properties of the pH marker, we investigated two different cell lines: epithelial A875 (human melanoma) and voluminous K562 (chronic myeloid leukemia), as well as normal fast-growing HEK293T cells (immortalized human embryonic kidney).

To understand the importance of using the R6G-spd-NBD conjugate, we used unmodified fluorophores R6G, NBD-spd, as a control. Figure 6a,b illustrate the fluorescent staining of A875 and K562 cells when exposed to NBD-spd, R6G, and R6G-spd-NBD, both in a conventional formulation and in a micellar one. The highest staining was achieved with the micellar form of the pH sensor R6G-spd-NBD, predominantly for cancer cells A875 and K562. While R6G and NBD-spd can also stain cancer cells effectively, they failed selectivity (selectivity indices of approximately 1.3–1.8), meaning they also stain normal cells (Figure 6c). At the same time, R6G-spd-NBD, in its micellar form, minimally penetrates HEK293T normal cells, indicating a very high level of efficiency with a selectivity index in about 10–12 (for non-micellar forms, the index is about 5–7 units) due to the simultaneous effect of pH sensitivity of the dye and the aspect of selective cell permeability for micelles.

R6G best visualizes volume-located leukemia cells K562 due to higher permeability. NBD-spd in micellar form penetrates better into adhesive cells A875 due to the increased adsorption of micelles on the cell surface. Therefore, the developed micellar formulations of pH marker R6G-spd-NBD are indeed able to selectively visualize cancer cells. The developed pH sensor and micellar formulations NBD-spd and R6G can be used for the selective imaging of melanoma cells and other tumors. 

As we continued to refine our approach to “Aikido micelles”, we became aware that they possess unique properties that make them both selective and pH-sensitive in the right location within specific cells. Additionally, it is noteworthy that by using the “smart” polymeric micelles based on the polyelectrolytes of different charges and structures, we open the possibility of regulating pH dependence of the fluorescence in the desired interval, which means they can be applied to the visualization of the variety of cell types, organelles, and other structures.

The comparison of the effectiveness of staining between cancer cells and normal cells is essential and relevant to correlate with intracellular pH levels. The intracellular pH values based on literature data [42,43,44,45] are as follows. Cancer cells: A875—pH 6.7 ± 0.2, K562—pH 7.1 ± 0.2. For normal cells (HEK293T), the pH is approximately 7.4–7.5.

According to the calibration dependences of R6G-spd-NBD fluorescence intensity on pH (Figure 5d), we could expect a difference between the fluorescence of cancer cells and normal ones.

**Figure 7:**
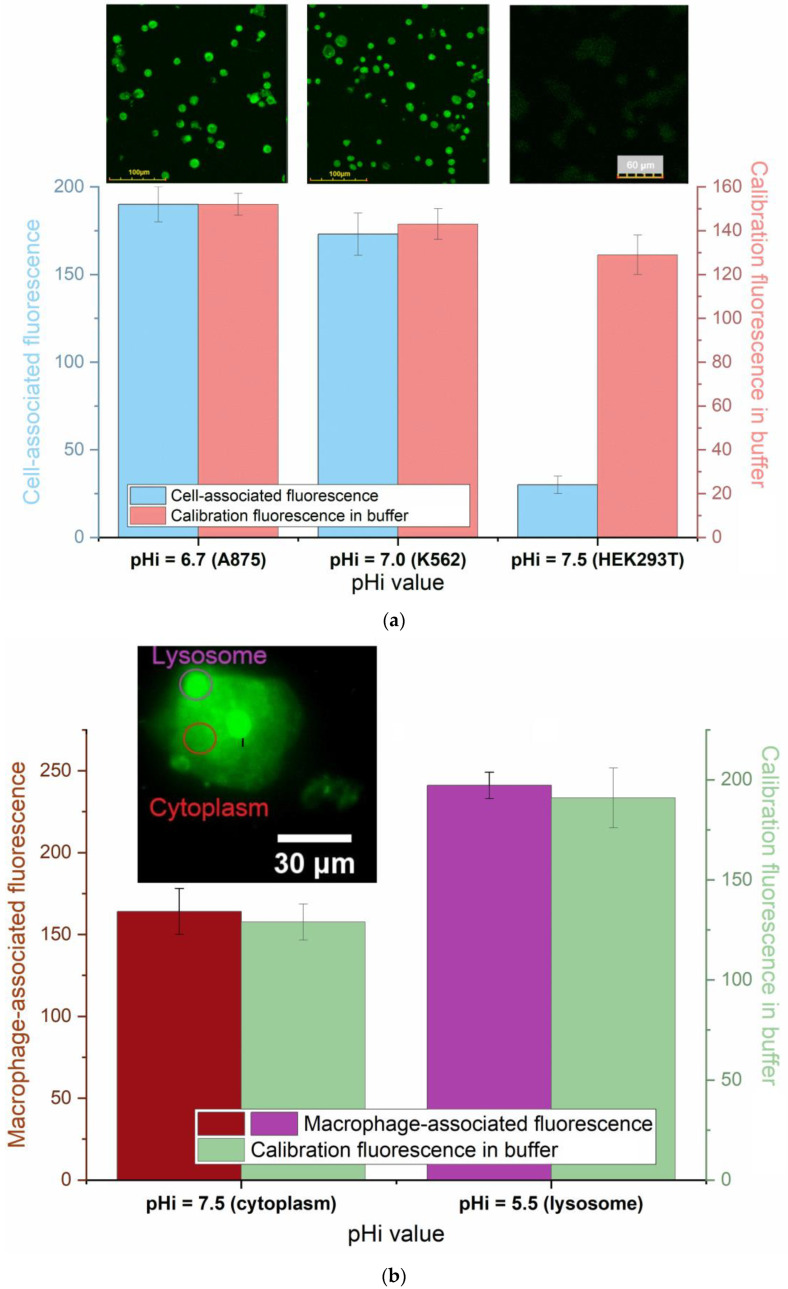
(**a**) Comparative fluorescence characteristics of the staining of A875, K562, and HEK293T cell cultures using the R6G-spd-NBD pH sensor loaded into Chit5-LA polymeric Aikido micelles. T = 37 °C. (**b**) Comparative fluorescence characteristics of the staining of macrophage cells (cytoplasm vs. lysosome) using the R6G-spd-NBD pH sensor loaded into Chit5-LA polymeric “Aikido micelles”. T = 37 °C.

Indeed, when examining HEK293T cells with pH values of 7.4–7.5 and cancer cultures with pH values pH < 7.0, a significant increase in the fluorescence in the case of cancer cells is observed, which indicates the pronounced sensitivity of the fluorescence intensity of R6G-spd-NBD to the intracellular pH. Figure 7a illustrates the comparative characteristics of staining various cell cultures using the R6G-spd-NBD pH sensor incorporated in the Chit5-LA polymeric micelles. The results demonstrate that the decrease in the intracellular pH enhances the fluorescent signal, which could be considered as a selective marker for cancer cells. However, the scope of application of these pH sensors is not limited but can potentially include visualization of cellular organelles (peroxisomes and lysosomes in macrophages) and bacterial cells.

The difference between the fluorescence curves obtained in buffer solutions and inside cells can be caused by two factors: (i) the pH sensitivity of the sensor and (ii) the selective permeability of micellar formulations to cancer cells. To demonstrate the impact of the pH sensitivity of the R6G-spd-NBD—sensor in the Chit5-LA polymeric micelles—on the fluorescence-enhancement effect observed, the control system was studied. The fluorescence in the lysosomes (pH 5.5) and cytoplasm (pH 7.4) of the macrophage were directly compared. Figure 7b illustrates the comparative characteristics of macrophage cell staining (cytoplasm and lysosome) using the R6G-spd-NBD pH sensor loaded into Chit5-LA polymer micelles. It can be seen that in the mildly acidic environment of the lysosome (pH 5.5), the pH sensor exhibits a glow of more than 50% greater intensity than in the neutral cytoplasmic environment, confirming the pH sensitivity of the presented R6G-spd-NBD sensor.

## 4. Conclusions

A significant challenge in modern biochemistry and biomedicine is to selectively visualize cancer cells. Selectivity in staining of cancer cells with a fluorescent dye can be achieved through pH dependence of the fluorescence within the range of 7.5 to 5.5. Fluorescent molecules can be made pH-sensitive in several ways using different mechanisms, including FRET, PeT, and structural changes in the fluorophore molecule. We selected the NBD and R6G pair as candidates for the creation of a pH-dependent fluorophore conjugate. To obtain the fluorescence pH sensitivity, we conjugated these two fluorophores using a cationic (pH dependent) linker spermidine (spd). As a result of four-stage synthesis using nucleophilic aromatic substitution reactions, setting and removing the protection of amino groups (via Schiff base), as well as the formation of an amide bond, a pH-sensitive fluorophore NBD-spd-R6G was obtained with a resultant yield of 50% (moderately soluble in water, well soluble in EtOH and DMSO). The selective visualization of cancer cells (A875, K562) was achieved via “smart micelles” based on chitosan grafted with lipoic acid residues. The micellar form of the NBD-spd-R6G fluorophore demonstrates a sharp ignition of fluorescence by 40% per 1 pH unit in the pH range from 7.5 to 5 due to polycationic properties of chitosan, resulting in the shift in the pH dependence of the fluorescence of the dye-conjugate. Additionally, due to micelles, the NBD-spd-R6G fluorophore actively penetrates cancer cells, and the ignition of fluorescence is observed, while micellar NBD-spd-R6G weakly accumulates and shows a low fluorescence signal in normal cells (HEK 293) due to the pH sensitivity of the dye. So, we demonstrated the potential of the developed micellar formulation for the selective visualization of cancer cells and targeting tumors compared to normal cells. The results obtained open the prospect of visualization of tumors in vivo, the staining of cancerous tissues for surgical purposes, or the selective delivery of drugs to tumors (with the ability to monitor their bio-distribution and pharmacokinetic characteristics).

## Figures and Tables

**Table 1:** Physico-chemical properties of the amphiphilic conjugate Chit5-LA and the “Aikido-micelles” based on it. T = 37 °C.

Property	Value
Chemical Designation	Chit5-LA (Chitosan—lipoic acid)
Chitosan Grafting Degree (by Glucosamine Units) *, %	20 ± 3
Average Mw of One Polymeric Structure Unit, kDa	5.8–6.5
Critical Micelle Concentration **, nM	7 ± 1
Hydrodynamic size of micelles, nm	Empty: 180 ± 40; Loaded with R6G-spd-NBD: 150 ± 30
Zeta potential, mV	+9 ± 1
Mass Fraction of Loaded Fluorophores, %	R6G—12, NBD-spd—15, R6G-spd-NBD—10

* Chitosan grafting degree was determined using spectrophotometric titration with 2,4,6-trinitrobenzenesulfonic acid [26]. ** Critical micelle concentrations were determined using the pyrene-probe technique [37].

**Table 2:** Parameters of pH sensor systems based on NBD-spd-R6G.

pH Sensor	pH Interval at Which the Fluorescence Intensity Changes Dramatically	Fluorescence Change in the Specified Range, % of the Signal/Unit pH	Fluorescence Ignition in the Target pH Range from 7.4 to 6 Units, %
NBD-spd-R6G at 22 °C	From 9.5 to 7.5	50 ± 2	1.7 ± 0.5
NBD-spd-R6G at 37 °C	From 9.5 to 7.5	53 ± 7	6 ± 1
NBD-spd-R6G in Aikido-micelles Chit5-LA at 22 °C	From 8 to 5	40 ± 8	28 ± 2
NBD-spd-R6G in Aikido-micelles Chit5-LA at 37 °C	From 8.5 to 3.5	37 ± 2	30 ± 2

## Data Availability

The data presented in this study are available in the main text.

## Supplementary Materials

The following supporting information can be downloaded at: https://www.mdpi.com/article/10.3390/pharmaceutics16081007/s1, Figure S1: ^1^H NMR spectra of (a) chitosan 5 kDa (Chit5) and lipoic acid-modified chitosan (Chit5-LA). T = 37 °C. Figure S2: Emission fluorescence spectra of poor-NBD-spd-R6G (synthesized without stages 1–2 with the formation of a nonstoichiometric conjugate) at 37 °C and different pHs. λ_exci_ = 460 nm.

## Abbreviations

Chit—chitosan; FRET—Förster resonance energy transfer; LA—lipoic acid; NBD—4-nitrobenzoxadiazole; NBD-Cl—7-chloro-4-nitrobenzoxadiazole; R6G—rhodamine 6G; spd—spermidine.
