# Supplementary material for: pH-Sensitive Fluorescent Marker Based on Rhodamine 6G Conjugate with Its FRET/PeT Pair in “Smart” Polymeric Micelles for Selective Imaging of Cancer Cells
Source: Pharmaceutics. 2024 Jul 30;16(8):1007. doi: 10.3390/pharmaceutics16081007 (PMC11360677; doi:10.3390/pharmaceutics16081007)
Supplement: Supplementary file 1 [file pharmaceutics-16-01007-s001.zip › pharmaceutics-3102589-supplementary.pdf]

pH-Sensitive Fluorescent Marker Based on Rhodamine 6G Conjugate with Its FRET/PeT Pair in "Smart" Polymeric Micelles for Selective Imaging of Cancer Cells

Igor D. Zlotnikov [1], Alexander A. Ezhov [2] and Elena V. Kudryashova [1,*]

[1] Faculty of Chemistry, Lomonosov Moscow State University, Leninskie Gory, 1/3, 119991 Moscow, Russia; zlotnikovid@my.msu.ru
[2] Faculty of Physics, Lomonosov Moscow State University, Leninskie Gory, 1/2, 119991 Moscow, Russia; alexander-ezhov@yandex.ru
* Correspondence: helenakoudriachova@yandex.ru

Figure S1. ^{1}H NMR spectra of (a) chitosan 5 kDa (Chit5) and lipoic acid modified chitosan (Chit5-LA). T = 37 °C.

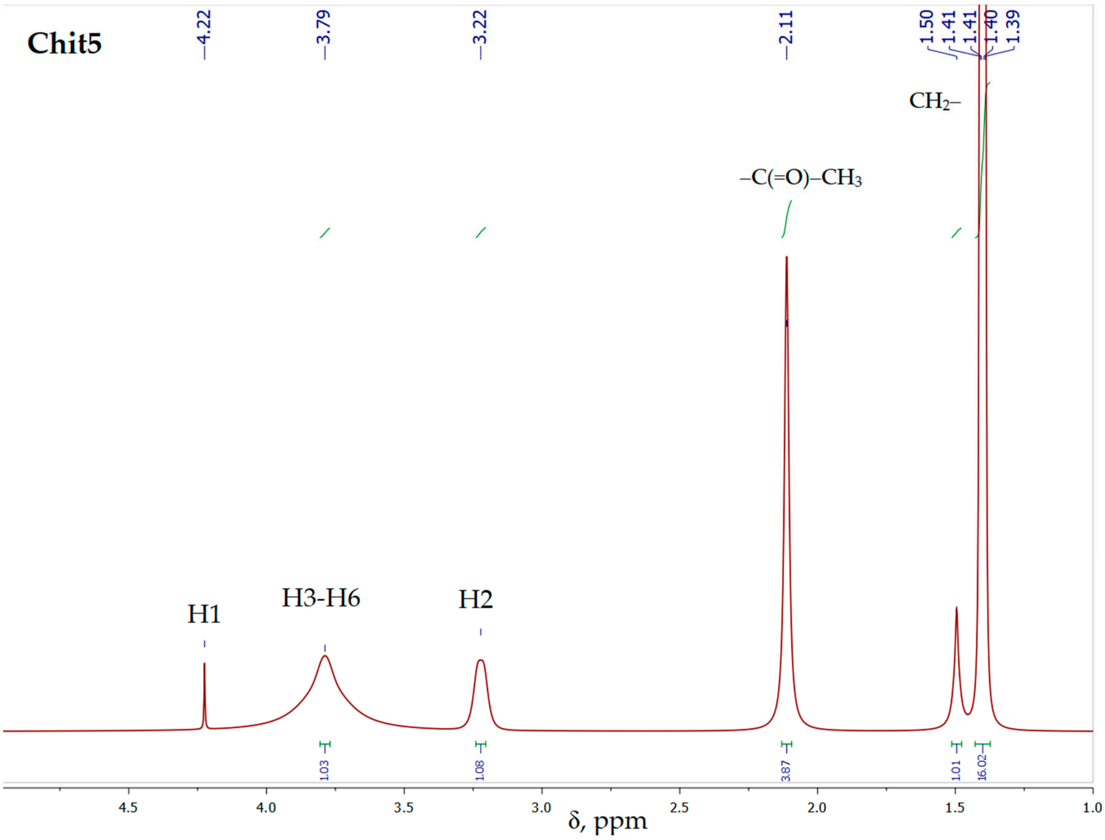

(**a**)

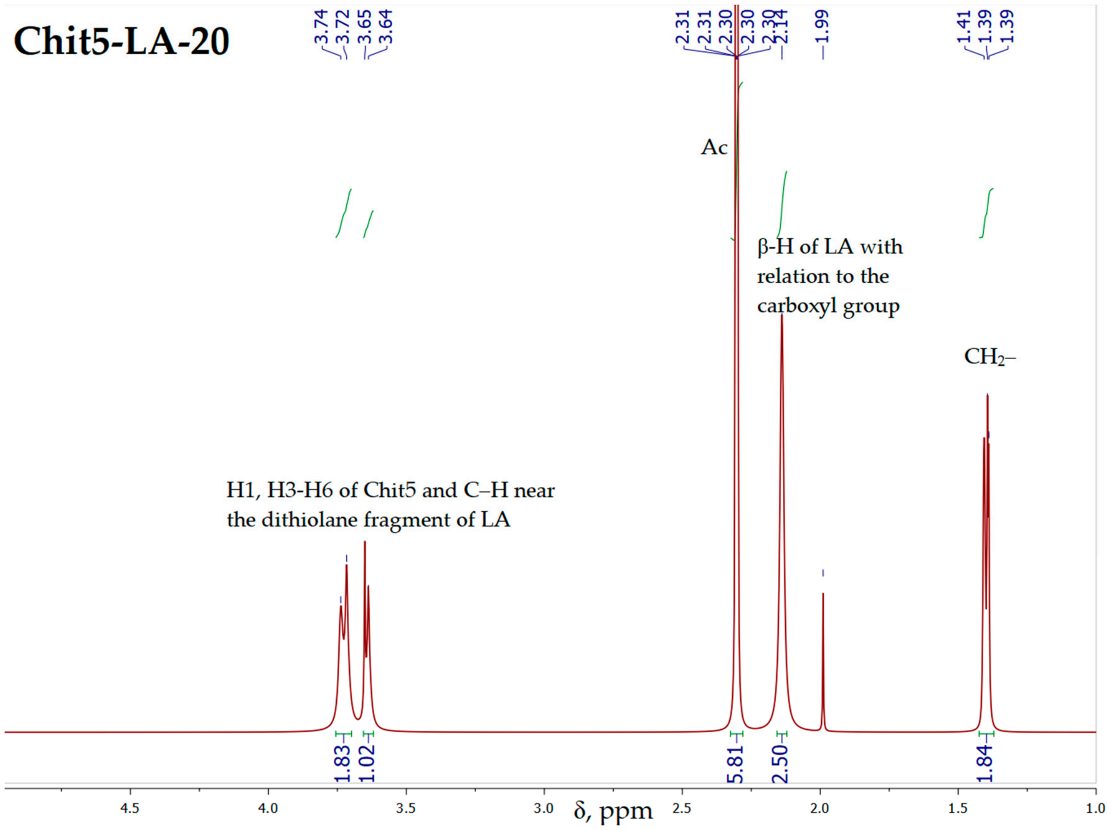

(**b**)

Figure S2. Emission fluorescence spectra of poor-NBD-spd-R6G (synthesized without stages 1-2 with the formation of a nonstoichiometric conjugate) at 37 °C and different pH. λ_{exci} = 460 nm.

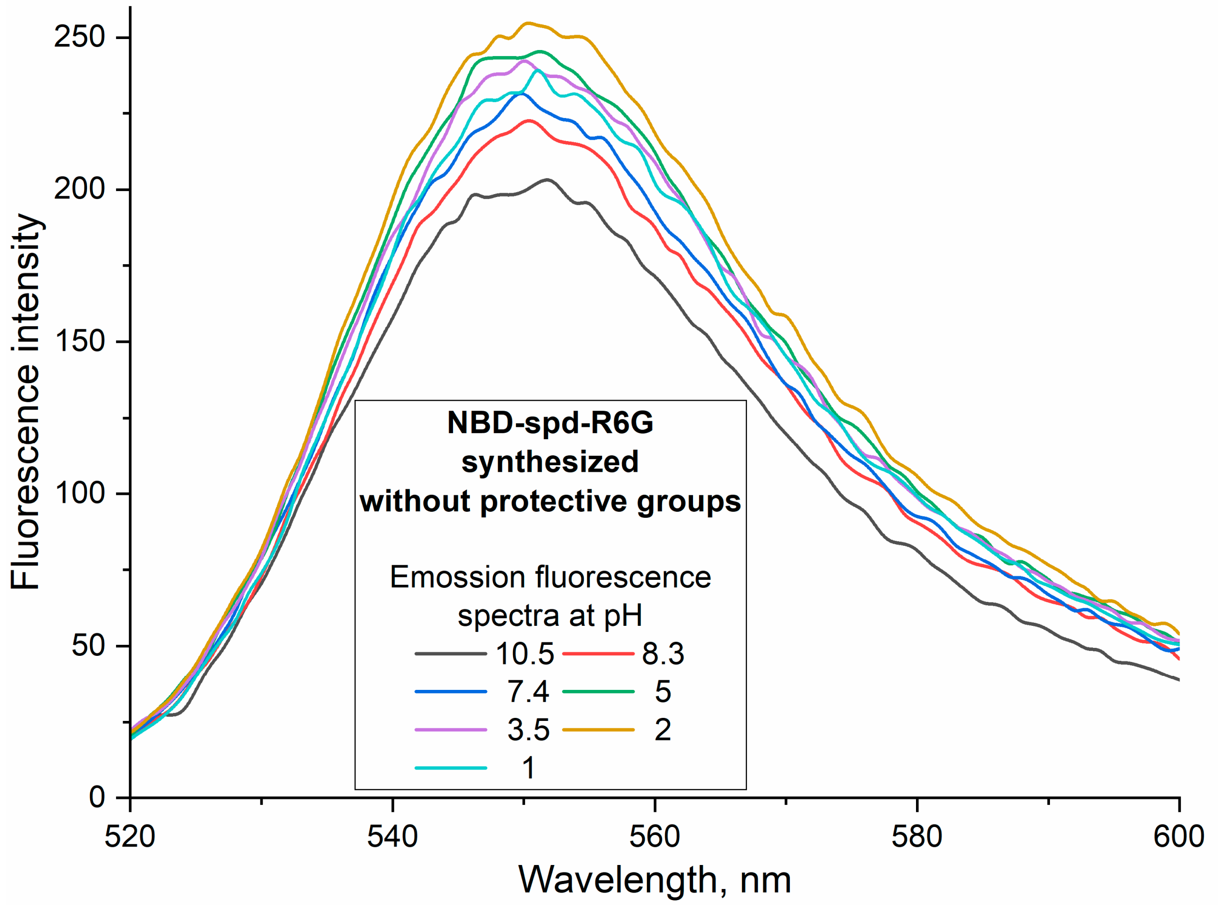
Fluorescence intensity
Wavelength, nm
0
50
100
150
200
250
520
540
560
580
600
NBD-spd-R6G
synthesized
without protective groups
Emossion fluorescence
spectra at pH
10.5
8.3
7.4
5
3.5
2
1
